# Overexpression of OqxAB and MacAB efflux pumps contributes to eravacycline resistance and heteroresistance in clinical isolates of *Klebsiella pneumoniae*

**DOI:** 10.1038/s41426-018-0141-y

**Published:** 2018-08-01

**Authors:** Jin-xin Zheng, Zhi-wei Lin, Xiang Sun, Wei-hong Lin, Zhong Chen, Yang Wu, Guo-bin Qi, Qi-wen Deng, Di Qu, Zhi-jian Yu

**Affiliations:** 1 Department of Infectious Diseases and the Key Lab of Endogenous Infection, Shenzhen Nanshan People’s Hospital, Shenzhen University School of Medicine, No 89, Taoyuan Road, Nanshan District, 518052 Shenzhen, China; 2 Key Laboratory of Medical Molecular Virology of Ministries of Education and Health, School of Basic Medical Science and Institutes of Biomedical Sciences, Shanghai Medical College of Fudan University, 200032 Shanghai, China

## Abstract

This study investigated the characteristics and mechanisms of eravacycline resistance and heteroresistance in clinical *Klebsiella pneumoniae* isolates. A total of 393 clinical *K*. *pneumoniae* isolates were collected and subjected to eravacycline and tigecycline MIC determinations using the agar dilution method. Eravacycline heteroresistance was assessed by a population analysis profile (PAP). The expression levels of efflux pumps and their regulators were determined by quantitative reverse-transcription PCR (qRT-PCR). This study identified 67 eravacycline-nonsusceptible isolates; among the extended-spectrum β-lactamase (ESBL)-positive isolates, eravacycline-nonsusceptible isolates were detected more frequently than tigecycline-nonsusceptible isolates (21.7% vs. 9.4%, *p* = 0.001). The study sample was observed to include 20 *K*. *pneumoniae* isolates with eravacycline heteroresistance. Compared to the reference strain, *oqxA* or *oqxB* overexpression was observed in nine eravacycline-nonsusceptible isolates (range, 35.64–309.02-fold) and 13 eravacycline-heteroresistant isolates (8.42–296.34-fold). The overexpression of *macA* or *macB* was detected in 12 eravacycline-heteroresistant isolates (3.23–28.35-fold). Overexpression of the efflux pump regulator gene *ramA* was observed in 11 eravacycline-nonsusceptible isolates (3.33–94.05-fold) and 18 eravacycline-heteroresistant isolates (3.89–571.70-fold). The eravacycline MICs were increased by one–fourfold by overexpression of *oqxAB* or *macAB* in three eravacycline-sensitive isolates. In conclusion, the overexpression of OqxAB and MacAB efflux pumps and the transcriptional regulator RamA were suggested to be involved in *K*. *pneumoniae* eravacycline resistance and heteroresistance.

## Introduction

Concerns regarding the Gram-negative pathogen *Klebsiella pneumoniae*, a member of the Enterobacteriaceae family, are growing worldwide due to the increasing incidence of severe infections, antibiotic-resistant strains, and reduced treatment efficacy^1^. Carbapenem-resistant Enterobacteriaceae are an emergent global health threat because carbapenems had previously been effective for eliminating multidrug-resistant Gram-negative bacterial infections^2^. In particular, increases in carbapenem-resistant *K*. *pneumoniae* (CR-Kp) frequencies worldwide are resulting in *K*. *pneumoniae* infections that are very difficult to treat and are thus associated with higher mortality rates^3^.

Tigecycline, the original member of the glycylcycline group of antibiotics, has been shown to have antimicrobial activity against CR-Kp in vitro, and thus, this antibiotic may be a last resort therapeutic option against CR-Kp infections^4^. However, cases of tigecycline-nonsusceptible *K*. *pneumoniae* (TNSKP) have emerged in hospitals with wide clinical application of tigecycline^5–7^. In recent years, TNSKP has been reported to occur in patients without prior exposure to tigecycline^8–10^. The mechanisms underlying tigecycline resistance are complex and not yet well understood. The overexpression of the efflux pumps AcrAB and OqxAB has been shown to play a crucial role in tigecycline resistance in *K*. *pneumoniae*^9,11^. Meanwhile, mutations in *ramR*, *acrR*, and *rpsJ* genes have also been reported to contribute to *K*. *pneumoniae* resistance to tigecycline^9,12^.

Eravacycline, previously known as TP-434, is a novel fluorocycline antibiotic with broad-spectrum activity against Gram-positive and Gram-negative aerobic and anaerobic pathogens in vitro^13^. Especially noteworthy is the observation that eravacycline has efficacy against several critical antimicrobial-resistant pathogens, such as methicillin-resistant *Staphylococcus aureus*, vancomycin-resistant enterococci, extended-spectrum β-lactamase (ESBL), carbapenemase-producing Enterobacteriaceae, and multidrug-resistant *Acinetobacter baumannii*^14–16^. Indeed, eravacycline has been reported to be two–fourfold more effective than tigecycline against common clinical Gram-positive and Gram-negative aerobic bacterial species^13^.

Like tigecycline, eravacycline can overcome most prevalent tetracycline-resistant mechanisms. Notably, Grossman et al. found that eravacycline efficacy was only slightly or undetectably affected by common tetracycline resistance factors, including efflux pumps (*tetA*, *tetB*, and *tetK*) and ribosomal protection protein (*tetM*) variants in *Escherichia coli*. Meanwhile, the antibacterial potencies of tigecycline and eravacycline were reduced by 4- to 16-fold in two nonisogenic *Propionibacterium acnes* isolates harboring a 16S rRNA gene (G1058C) mutation compared with that of the wild-type control strain^15^. The MIC for eravacycline in *adeB*-hyperexpressing *A*. *baumannii* was shown to be reduced by eightfold by disrupting the gene *adeB*^17^. However, the traits and mechanisms of eravacycline resistance among clinical *K*. *pneumoniae* isolates, especially TNSKP isolates, have yet to be clarified. Thus, the aim of the present study was to explore the characteristics and mechanisms of eravacycline resistance among clinical *K*. *pneumoniae* isolates.

## Results

### Tigecycline and eravacycline susceptibilities among clinical *K*. *pneumoniae* isolates

As shown in Table 1, tigecycline-nonsusceptible isolates of *K*. *pneumoniae* were similarly represented between ESBL-positive and -negative strains, as well as between carbapenem-resistant and -susceptible strains. Eravacycline susceptibility was less common among ESBL-positive strains than among ESBL-negative strains (*P* < 0.05). Among the ESBL-positive isolates, eravacycline nonsusceptibility was more common than tigecycline nonsusceptibility (21.7% vs. 9.4%, *P* = 0.001).

**Table 1:** Tigecycline and eravacycline susceptibility characteristics among 393 clinical *K*. *pneumoniae* isolates

Characteristic	Tigecycline MIC (mg/L)		Eravacycline MIC (mg/L)	
	≤2	≥4	≤2	≥4
ESBL^a^				
Positive (*n* = 203)	184 (90.6)	19 (9.4)	159 (78.3)	44 (21.7)^b, c^
Negative (*n* = 145)	131 (90.3)	14 (9.7)	129 (89.0)	16 (11.0)
CR-Kp				
Positive (*n* = 45)	39 (86.7)	6 (13.3)	37 (82.2)	8 (17.8)
Negative (*n* = 348)	316 (90.8)	32 (9.2)	289 (83.1)	59 (16.9)
Total (*n* = 393)	355 (90.3)	38 (9.7)	326 (83.0)	67 (17.0)

*Note*: Data shown as *n* (%)

*ESBL* extended-spectrum β-lactamase, *CR-Kp* carbapenem-resistant *K. pneumoniae*

^a^CR-Kp isolates excluded

^b^ESBL: positive vs. negative, *P* < 0.05

^c^Among ESBL**-**positive isolates, eravacycline MIC ≥4 vs. tigecycline MIC ≥4, *P* < 0.05

### Effects of efflux pump inhibitor (EPI) and ribosomal protein gene mutations on *K*. *pneumoniae* MICs

To investigate the mechanisms of eravacycline resistance in *K*. *pneumonia*, 37 clinical *K*. *pneumoniae* isolates were selected for further study, including the tigecycline- or eravacycline-susceptible or nonsusceptible isolates. To explore the differences between eravacycline and tigecycline resistance, these 37 isolates were divided into three groups, those that had MICs of tigecycline that were <, =, or > the MICs of eravacycline. The effects of the EPI Phe-Arg-β-naphthylamide (PAβN) on tigecycline and eravacycline MICs are reported in Table 2. Notably, among 25 tigecycline- and/or eravacycline-nonsusceptible isolates (MIC ≥4 mg/L), six isolates showed a 16-fold decrease, 10 showed an eightfold decrease, eight showed a fourfold decrease, and one isolate showed a twofold decrease in tigecycline and/or eravacycline MICs in the presence of PAβN (50 mg/L). However, among 12 tigecycline- and eravacycline-susceptible isolates (MIC ≤ 2 mg/L), three showed a fourfold decrease and six showed a twofold decrease in tigecycline and/or eravacycline MICs in the presence of PAβN (50 mg/L).

**Table 2:** Tigecycline and eravacycline MICs in the absence or presence of PAβN, and ribosomal protein gene mutations in 37 clinical *K. pneumoniae* isolates

Isolate	MIC (mg/L)				Local repressor gene mutation(s)		
	Tig	Tig + PAβN	Era	Era + PAβN	*acrR*	*rpsJ*	*ramR*
EKP194	8	1	4	1	Y170Stop	—	N131Y
CRKP10	8	0.5	4	0.5	—	—	—
EKP19	4	2	2	1	P82L	—	T43K
EKP48	4	1	2	1	T160I	—	—
LBKP25	2	0.5	0.5	0.5	—	—	—
EKP176	1	0.5	0.5	0.5	C148R	—	—
EKP185	1	0.5	0.25	0.25	—	—	—
EKP201	1	0.5	0.25	0.25	—	—	—
CRKP14	1	0.5	0.5	0.5	—	—	—
CRKP29	1	1	0.5	0.5	—	—	—
EKP192	16	4	16	2	—	—	—
EKP195	16	1	16	1	—	—	—
EKP154	8	2	8	1	—	—	—
CRKP6	8	1	8	0.5	—	—	—
CRKP8	8	1	8	0.5	—	—	—
CRKP21	8	1	8	0.5	—	—	—
EKP135	4	0.5	4	0.5	L187P	—	—
LBKP50	4	0.5	4	0.5	—	—	—
EKP122	2	1	2	1	—	—	—
LBKP84	2	1	2	0.5	—	—	—
EKP88	1	1	1	1	Y114F, V165I	—	H186N
EKP100	1	1	1	1	—	—	—
EKP178	8	2	16	1	—	—	A22P
EKP86	4	2	8	1	—	—	—
EKP56	2	1	8	1	C148R	—	—
EKP108	2	2	8	1	—	—	—
EKP217	2	2	8	1	—	—	—
EKP58	1	1	4	1	—	—	—
EKP90	2	0.5	4	0.5	—	—	—
LBKP103	2	1	4	1	—	—	I141T
EKP232	0.5	0.5	4	1	—	—	—
EKP1	1	1	4	1	—	—	—
EKP99	1	0.5	4	1	—	—	—
LBKP102	2	1	4	1	—	—	—
LBKP61	2	1	4	1	—	—	—
EKP3	0.5	0.5	2	0.5	—	—	—
EKP42	0.5	0.5	2	1	—	—	—

*Tig* tigecycline, *Era* eravacycline, *PAβN* Phe-Arg-b-naphthylamide (50 mg/L)

Mutations in *acrR* were observed in seven of the above 37 clinical *K*. *pneumoniae* isolates and in five of the 25 tigecycline- and/or eravacycline-nonsusceptible isolates (Table 2). Mutations in *ramR* were detected in five of these 37 clinical *K*. *pneumoniae* isolates and in three of the 25 tigecycline- and/or eravacycline-nonsusceptible isolates. Three isolates were observed with both *ramR* and *acrR* mutations, two of which were tigecycline- and/or eravacycline-nonsusceptible and had MICs comparable to other nonsusceptible isolates. None of the isolates in this study had *rpsJ* mutations.

Furthermore, we detected these ribosomal protein gene mutations in 47 tigecycline- and eravacycline-sensitive clinical *K*. *pneumonia* isolates. Mutations in *acrR* or *ramR* were also observed in these eravacycline-sensitive isolates, but were only detected in four isolates and were different from those mutations detected in eravacycline-nonsusceptible isolates (Table S1).

### Expression of efflux pump and regulator genes in clinical *K*. *pneumoniae* isolates

**Fig. 1:**
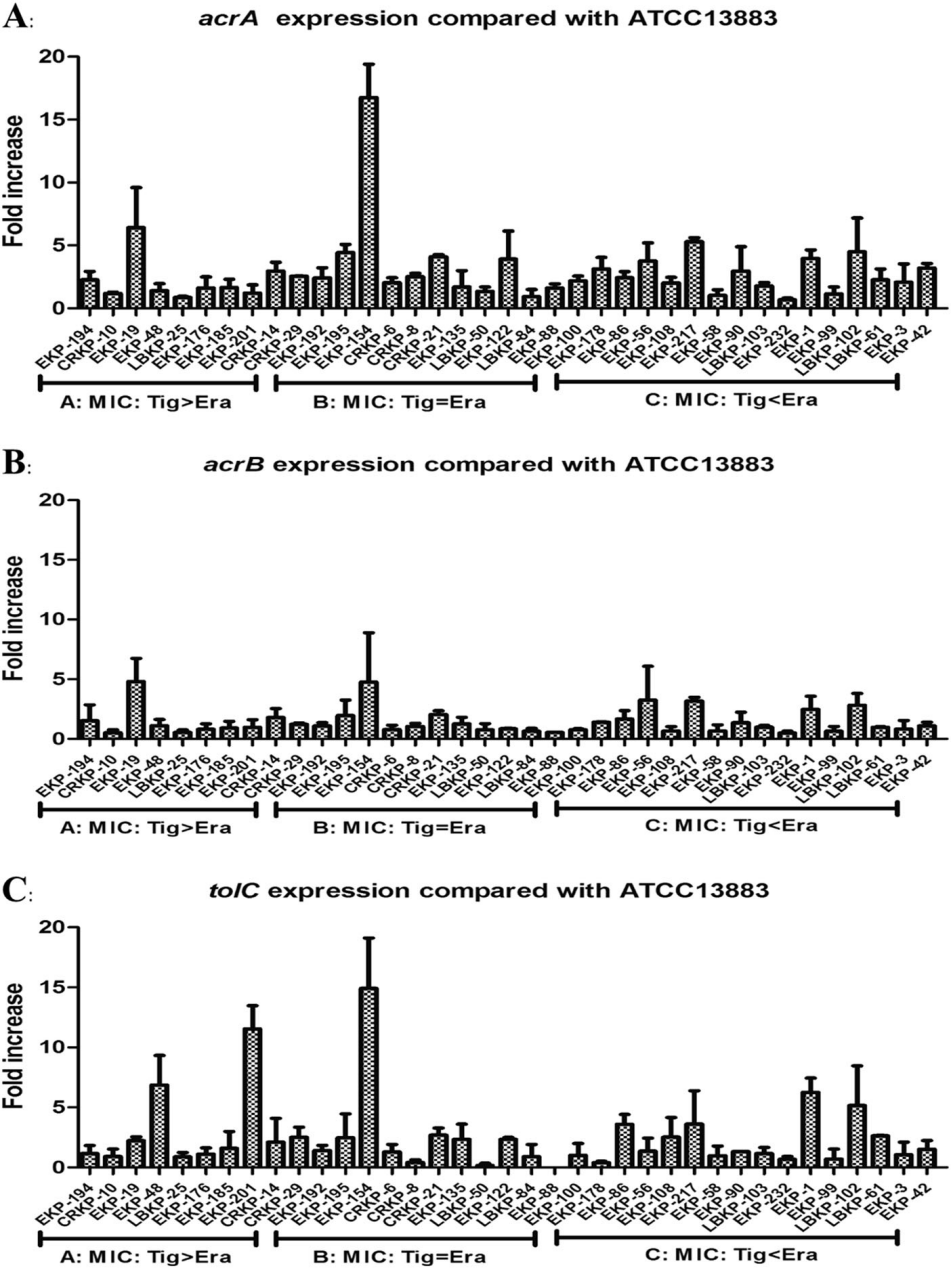
Relative gene expression, expressed as fold change, of the *acrAB-tolC* efflux pump in 37 clinical *K. pneumoniae* isolates. Expression levels were detected by qRT-PCR, with tigecycline-susceptible *K*. *pneumoniae*, ATCC 13883 used as the reference strain (expression = 1)

**Fig. 2:**
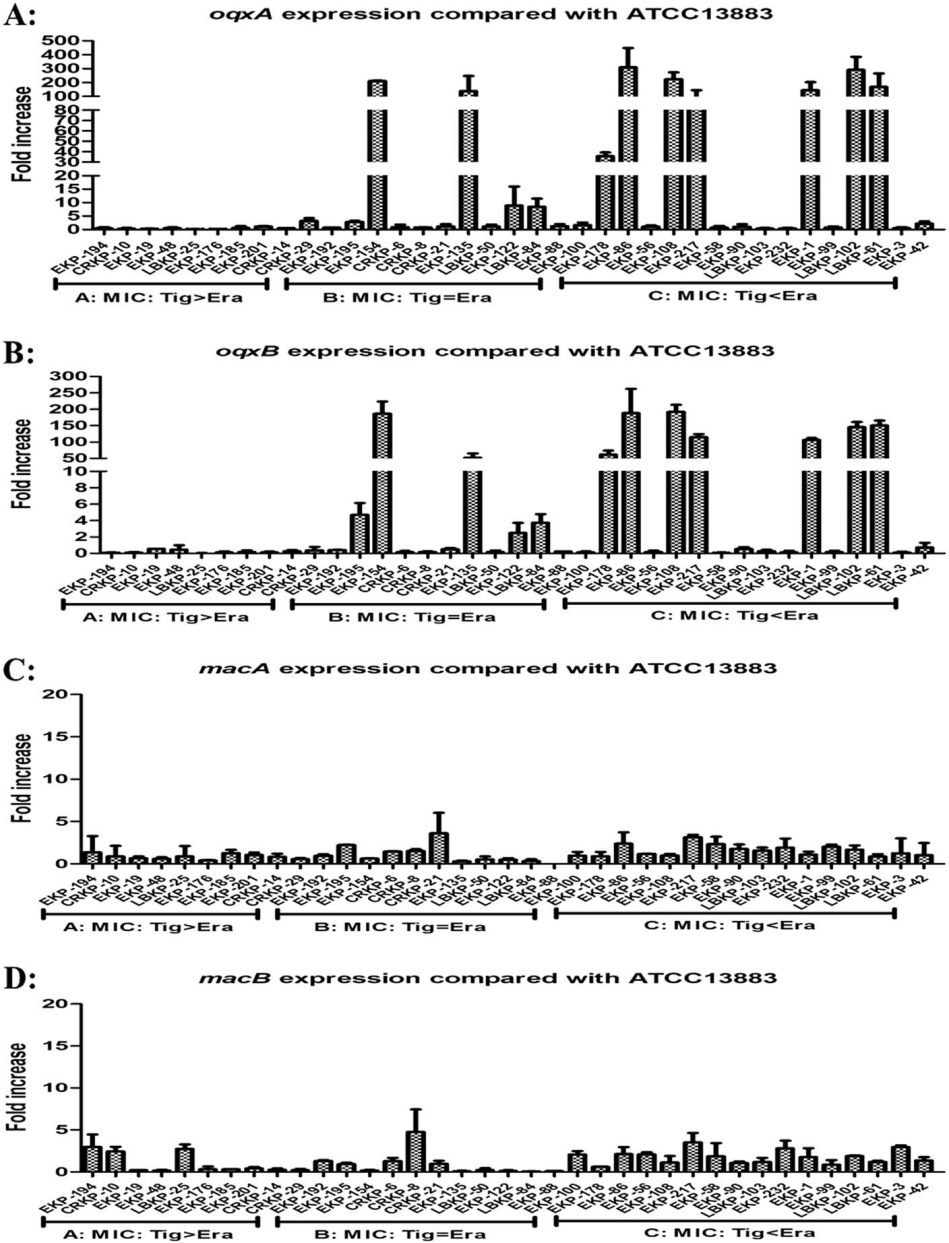
Relative gene expression, expressed as fold change, of *oqxAB* and *macAB* efflux pumps in 37 clinical *K. pneumoniae* isolates. Expression levels were detected by qRT-PCR, with tigecycline-susceptible *K*. *pneumoniae* ATCC 13883 used as the reference strain (expression = 1)

Our qRT-PCR experiments indicated that only 6/37 isolates overexpressed the AcrAB–TolC pathway genes *acrA* and/or *tolC* (>fivefold greater than the tigecycline-susceptible *K*. *pneumoniae* ATCC 13883 reference strain), with a maximum value detected as 16.77-fold compared with the reference strain (Fig. 1). Interestingly, 11/37 isolates were observed to overexpress *oqxA* or *oqxB* (range, 8.46–309.02-fold compared with the reference strain levels), with a maximum value of 309.02-fold (Fig. 2). Among these 11 isolates, nine exhibited a significant overexpression of *oqxAB* (35.64–309.02-fold) and all nine isolates were tigecycline- and/or eravacycline-nonsusceptible, with observed eravacycline MICs greater than or equal to the tigecycline MICs in all nine isolates. Two isolates were observed to overexpress *acrF* (6.55- and 19.74-fold) (Fig. S1). Only 11/37 isolates were observed to overexpress *ramA* (3.33–94.05-fold), and only 8/37 isolates were observed to overexpress *soxS* (2.02–11.39-fold) (Fig. S2).

### Efflux pump activity and ribosomal protein gene mutations in eravacycline-heteroresistant isolates

A population analysis profile (PAP) of eravacycline susceptibility (MIC <2 mg/L) among clinical *K*. *pneumoniae* isolates indicated that 20 isolates could be classified as having heteroresistance to eravacycline. PAβN (50 mg/L) exposure resulted in eravacycline resistance reductions that were modest (four- to eightfold reduction) in 5/20 heteroresistant isolates, moderate (16- to 32-fold) in 7/20 heteroresistant isolates, pronounced (64-fold) in 5/20 heteroresistant isolates, and very pronounced (128-fold) in 3/20 heteroresistant isolates (Table 3). These results show that a pharmacological EPI can suppress efflux pump activity in heteroresistant isolates beyond that exhibited in the eravacycline-resistant isolates of *K*. *pneumoniae*.

**Table 3:** Eravacycline MICs in the absence or presence of PAβN and ribosomal protein gene mutations in 20 eravacycline-heteroresistant clinical *K. pneumoniae* isolates

Isolate	MIC (mg/L)		Mutations of ribosomal protein genes		
	Era	Era + PAβN	*acrR*	*rpsJ*	*ramR*
EKP155-2	16	4			A61P
EKP162-1	8	2			
EKP82-2	16	2			
EKP100-1	16	2			
EKP119-1	8	1	Y114F, V165I		R120P, H186N
EKP92-1	16	1	Y114F, V165I		H186N
EKP66-1	16	1			
EKP28-1	8	0.5			T119P
EKP57-1	8	0.5			
EKP83-2	16	0.5			
EKP165-1	16	0.5			
EKP11-1	8	0.25			I106F
EKP109-1	16	0.25			M109R
EKP97-1	16	0.25			
EKP220-1	16	0.25			R35S, I106L
EKP55-1	16	0.25			
EKP229-1	8	0.125			
EKP60-1	16	0.125			A159E
EKP17-1	16	0.125	A20V		
EKP129-1	16	0.125			

*Era* eravacycline, *PaβN* Phe-Arg-b-naphthylamide (50 mg/L)

Only 3/20 eravacycline-heteroresistant isolates were observed to have *acrR* mutations, whereas 8/20 eravacycline-heteroresistant isolates were observed to have *ramR* mutations (Table 3). None of the 20 eravacycline-heteroresistant isolates had an *rpsJ* mutation.

### Pump and regulator gene expression in eravacycline-heteroresistant isolates

**Fig. 3:**
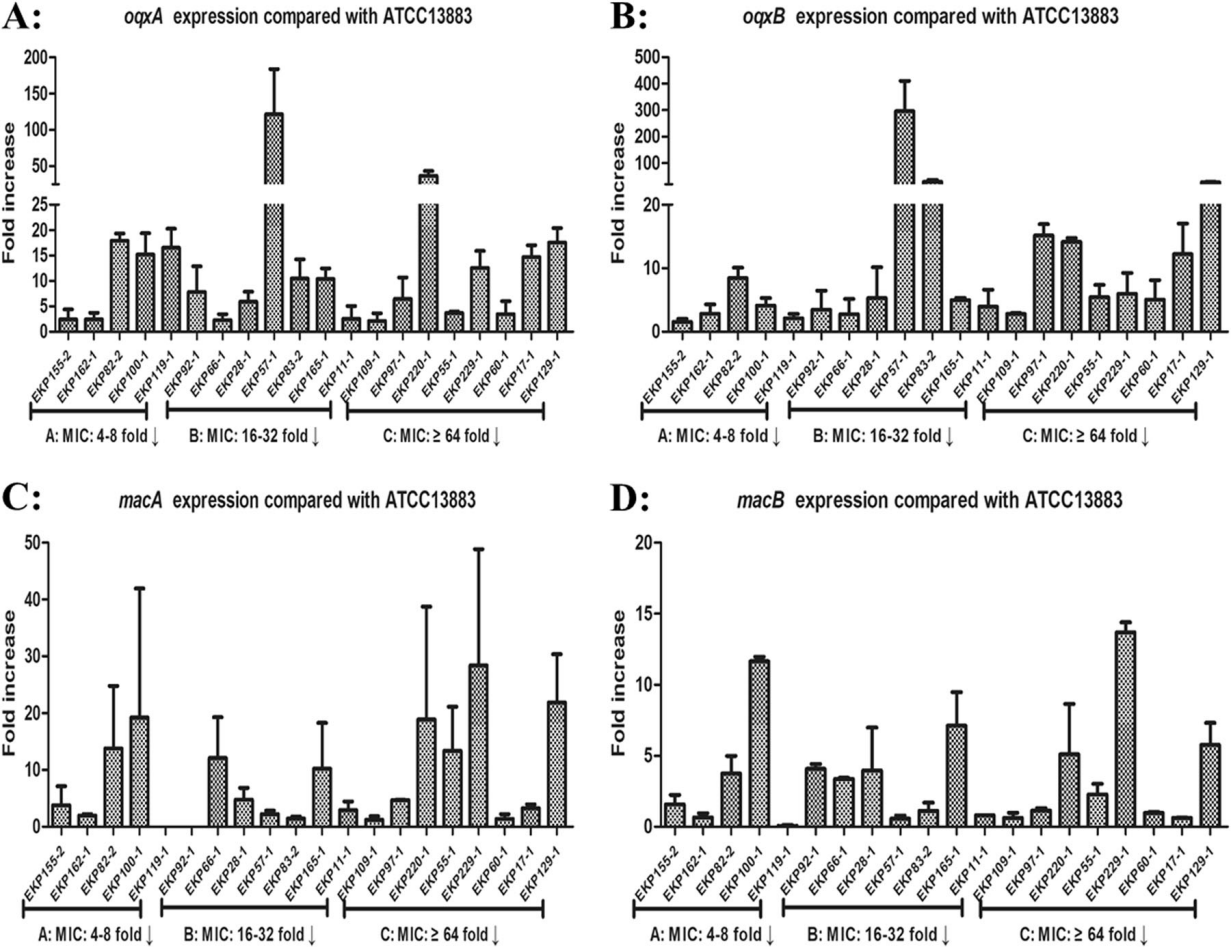
Relative gene expression, expressed as fold change, of *oqxAB* and *macAB* efflux pumps in 20 clinical eravacycline-heteroresistant *K. pneumoniae* isolates. Expression levels were detected by qRT-PCR, with tigecycline-susceptible *K*. *pneumoniae* ATCC 13883 used as the reference strain (expression = 1). MIC: 4–8-fold↓, PAβN reduced eravacycline MICs by 4–8-fold compared to eravacycline alone

Among the 20 eravacycline-heteroresistant isolates studied, *oqxA* (15.61 ± 22.23-fold) or *oqxB* (22.63 ± 64.90-fold) was significantly overexpressed, relative to the reference strain, in 13/20 eravacycline-heteroresistant isolates (Fig. 3). Overexpression of *macA* (8.26 ± 8.43-fold) or *macB* (3.44 ± 4.59-fold), relative to the reference strain, was observed in 12/20 eravacycline-heteroresistant isolates (Fig. 3). While only seven eravacycline-heteroresistant isolates were observed to overexpress *acrA*, *acrB*, or *tolC* (≥fivefold compared with the reference strain *K*. *pneumoniae* ATCC 13883), the mean fold differences in the expression of these genes relative to the reference strain were 4.36 ± 2.84, 3.48 ± 1.72, and 2.91 ± 2.76, respectively (Fig S3). Regarding efflux pump regulator genes, 18/20 eravacycline-heteroresistant isolates were observed to overexpress *ramA* relative to the reference strain (mean, 147.65 ± 164.40-fold; maximum, 571.70-fold; Fig S4), and the overexpression of *ramA* in these eravacycline-heteroresistant isolates exceeded that observed in eravacycline-resistant isolates (14.58 ± 30.15-fold). Notably, 20 eravacycline-heteroresistant isolates were observed to include eight isolates with *ramR* mutations (40.0%), while only 3/23 eravacycline-resistant isolates were observed to have *ramR* mutations (13.0%) (Tables 2 and 3).

### Mutations in OqxAB and MacAB efflux pumps

Mutations in *oqxAB* and *macAB* genes in the assayed clinical *K*. *pneumoniae* isolates overexpressing *oqxAB* or *macAB* (overexpressed >threefold greater than the ATCC 13883 reference strain) were identified. Mutations in OqxAB and MacAB efflux pumps were rarely reported from previous studies. In this study, only two eravacycline-heteroresistant isolates with *oqxAB* mutations (EKP82-2 and EKP119-1) and one isolate with a *macB* mutation (EKP82-2) were identified, which were not present among those eravacycline-resistant isolates (Table S2 and S3).

### OqxAB and MacAB efflux pumps associated with eravacycline resistance were tested in an overexpression experiment

**Fig. 4:**
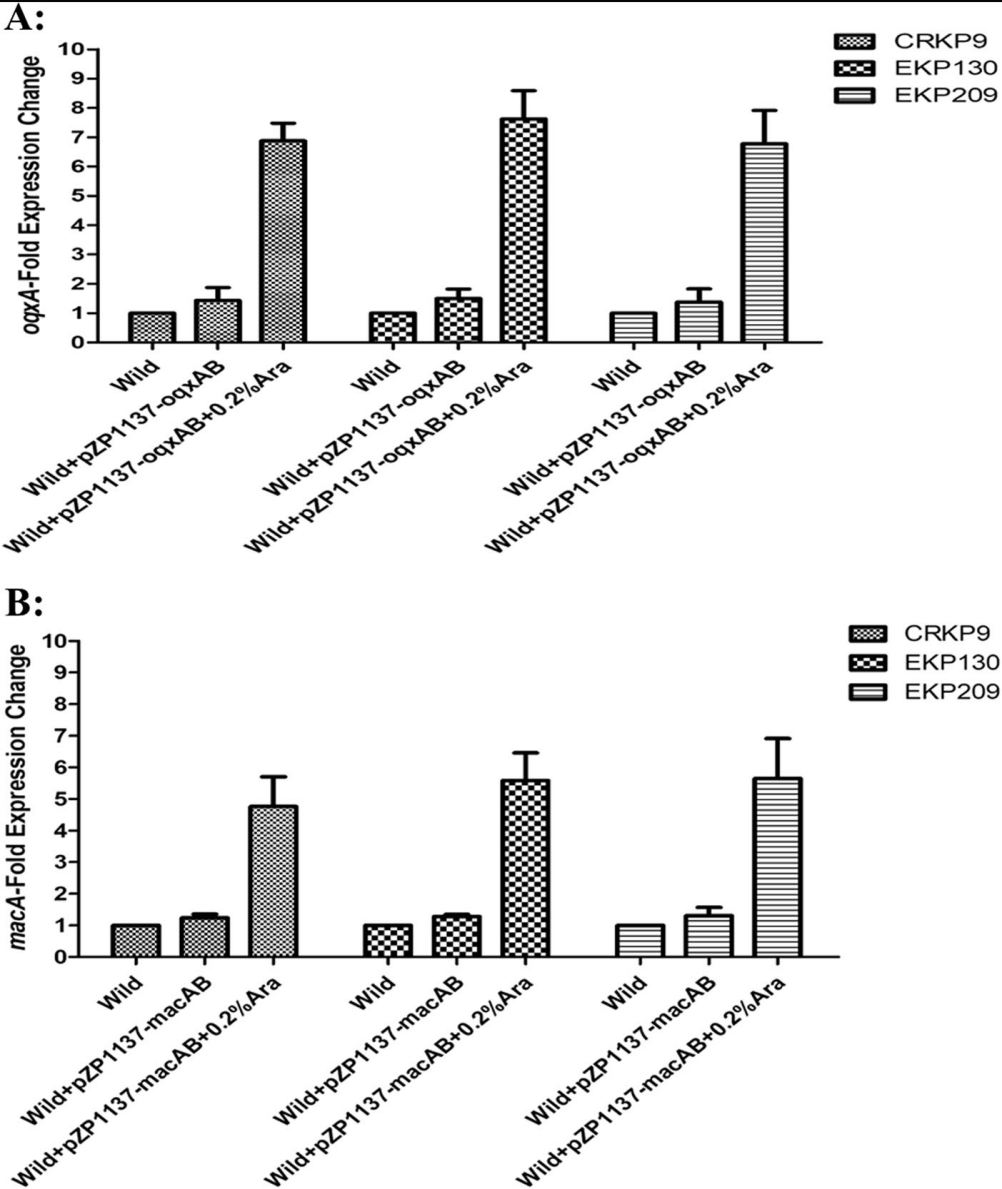
Overexpression of OqxAB and MacAB in three eravacycline-sensitive clinical *K. pneumoniae* isolates. The expression levels of *oqxA* (**a**) or *macA* (**b**) was determined by qRT-PCR. The wild-type strain of these three isolates were used as the reference strains (expression = 1.0)

To confirm the roles of OqxAB and MacAB efflux pumps in eravacycline resistance of *K*. *pneumoniae*, the overexpression of *oqxAB* and *macAB* in three eravacycline-sensitive isolates was conducted. As Fig. 4 shows the expression levels of *oqxA* increased 6.77–7.63-fold and that of *macA* increased 4.76–5.65-fold, following a 0.2% arabinose (Ara) induction. Interestingly, the eravacycline MICs of these three eravacycline-sensitive isolates increased one–fourfold following the 0.2% Ara induction, relative to controls not exposed to Ara (Table 4).

**Table 4:** Overexpression of OqxAB or MacAB in three eravacycline-sensitive clinical *K. pneumoniae* isolates

Isolates	Eravacycline MIC (mg/L)		
	CRKP9	EKP130	EKP209
Wild	0.25	0.5	0.25
Wild + pZP1137-*oqxAB*	0.25	0.5	0.25
Wild + pZP1137-*oqxAB* + 0.2%Ara	1	2	1
Wild + pZP1137-*macAB*	0.25	0.5	0.25
Wild + pZP1137-*macAB* + 0.2%Ara	0.5	2	0.5

*Ara* arabinose

## Discussion

In previous studies, researchers observed that eravacycline had a more powerful antibacterial effect than tigecycline against clinically common aerobic bacterial species^13,15^. However, the results of this study showed that eravacycline-nonsusceptible clinical *K*. *pneumoniae* isolates were encountered more frequently than tigecycline-nonsusceptible isolates, especially among ESBL-positive isolates. The occurrence of antibiotic resistance varies regionally. Until now, there have been no reports of eravacycline susceptibility in *K*. *pneumoniae* from China. We believe that the *K*. *pneumoniae* isolates from different regions provide an explanation for different results, but this issue needs additional study in China for confirmation. In the present study, the overexpression of several antibiotic resistance-related genes, including genes encoding proteins that constitute the resistance-nodulation-cell division (RND)-type efflux pump AcrAB, the quinolone/olaquindox efflux pump OqxAB, and the transcriptional activators RamA and SoxS, was observed among the examined eravacycline-nonsusceptible isolates. Imipenem, meropenem, and colistin heteroresistances have been documented previously in carbapenemase-producing isolates of *K*. *pneumoniae*^18–21^. To the best of our knowledge, this is the first study to clinically document *K*. *pneumoniae* isolates with eravacycline heteroresistance.

The RND-type efflux pump, AcrAB-TolC, has been shown to play an important role in Enterobacteriaceae antimicrobial resistance, especially tigecycline resistance in *K*. *pneumoniae*^4,9,22,23^. Quinolone/olaquindox efflux pump, OqxAB, was also associated recently with tigecycline resistance in *K*. *pneumoniae*^8,11^. The overexpression of AcrAB-TolC and OqxAB in clinical *K*. *pneumoniae* isolates without a history of tigecycline exposure may be due to exposure to other antimicrobials that are transported by the same efflux pumps^8–12^. The eravacycline-resistant and eravacycline-heteroresistant *K*. *pneumoniae* isolates assayed in this study had not been exposed to tigecycline or eravacycline, but their overexpression of AcrAB-TolC and/or OqxAB is consistent with the possibility of their exposure to other compounds transported by these pumps. Although the overexpression of both AcrAB-TolC and OqxAB have previously been implicated in tigecycline resistance in *K*. *pneumoniae*; in this study, we observed a more pronounced OqxAB overexpression relative to that of AcrAB-TolC in eravacycline-resistant and -heteroresistant isolates of *K*. *pneumoniae*. Similar to previous studies, we observed that the MICs of some eravacycline-resistant and -heteroresistant isolates were inhibited significantly by PAβN, despite the low-level expression of AcrAB-TolC or OqxAB^9^. Eravacycline resistance in these *K*. *pneumoniae* isolates may be due to efflux pumps other than AcrAB-TolC or OqxAB.

The overexpression of the RND-type efflux pump AcrEF was shown to be associated with fluoroquinolone resistance in *Salmonella enterica* serovar Typhimurium^24^. A recent study by Zhang et al.^25^ observed that AcrEF upregulation contributes to quinolone resistance development in *E*. *coli*. Two eravacycline-resistant *K*. *pneumoniae* isolates (EKP195 and EKP56) that overexpressed AcrF in the present study provide the first report of the involvement of AcrF in *K*. *pneumoniae* tetracycline resistance.

This study provides the first evidence implicating the overexpression of the periplasmic adapter MacAB in eravacycline resistance and heteroresistance in clinical *K*. *pneumoniae* isolates. The MacAB-TolC pump assembly, which is now the best-studied bacterial ABC drug exporter, was demonstrated in *E*. *coli* to be a cell envelope-spanning transmembrane transporter that actively extrudes the substrates, including macrolide antibiotics and polypeptide virulence factors^26,27^. Our observation of high levels of *macA* expression in 12 eravacycline-heteroresistant isolates indicates that the MacAB-TolC multidrug efflux pump may, like OqxAB, play an important role in eravacycline heteroresistance in *K*. *pneumoniae*. Previous studies have indicated that tigecycline resistance in *K*. *pneumonia* is primarily due to the overexpression of AcrAB efflux pump. However, the results of this study showed that the OqxAB and MacAB efflux pumps play a more important role than AcrAB in the development of eravacycline resistance in *K*. *pneumonia*. The reason for this difference may be that the C-7 and C-9 of the tetracycline core D-ring of eravacycline are notably different from those in tigecycline, although the two antibacterials belong to the tetracycline family^13^.

The expression of AcrAB-TolC efflux pump can be modulated by several transcriptional regulators, including those encoded by *ramA*, *marA*, *rarA*, *soxS*, and *robA*^4,22,23^. In the present study, we observed the overexpression of transcriptional regulator *ramA* in our eravacycline-resistant and -heteroresistant *K*. *pneumoniae* isolates without the overexpression of AcrAB-TolC efflux pump-encoding genes. Interestingly, the overexpression of OqxAB and MacAB-TolC efflux pump-encoding genes was observed in eravacycline-resistant and -heteroresistant *K*. *pneumoniae* isolates. The efflux pump OqxAB is activated by the transcriptional regulators RamA and RarA^8,28^. The observed overexpression of OqxAB in this study may have been related to the upregulated expression of RamA, rather than being attributable to RarA or other transcriptional regulators. The *ramR* gene is located upstream of *ramA*, and *ramR* is a negative regulator of *ramA*. Thus, diverse *ramR* mutations can lead to the upregulation of *ramA* expression and subsequently contribute to reduced antibiotic susceptibility^9,28^. In this study, the eravacycline-heteroresistant isolates with higher *ramA* expression than those in eravacycline-resistant isolates may be due to the heteroresistant isolates having more *ramR* mutations. However, among some eravacycline-resistant (EKP195, EKP154, CRKP21, and EKP217) and -heteroresistant (EKP162-1, EKP82-2, EKP100-1, EKP66-1, EKP57-1, EKP55-1, EKP229-1, EKP17-1, and EKP129-1) isolates, we observed elevated *ramA* expression in the absence of any *ramR* mutations. Thus, the mechanisms responsible for high *ramA* expression may due to other unknown transcriptional regulators in those isolates and require further investigation.

Nonetheless, the above-reported efflux pumps cannot explain the observed eravacycline resistance observed in four isolates (EKP192, CRKP6, CRKP21, and EKP56), whose MICs were inhibited significantly by PAβN despite the low-level expression of efflux pump genes. Therefore, the underlying mechanisms for these eravacycline-resistant isolates need to be further studied. The role of the MacAB-TolC multidrug efflux pump in *K*. *pneumoniae* resistance to eravacycline also requires further study.

In conclusion, the results of this study implicate the overexpression of OqxAB, but not AcrAB-TolC, as well as the overexpression of the transcriptional regulator RamA in *K*. *pneumoniae* eravacycline resistance and heteroresistance. Moreover, the present data suggest that the MacAB-TolC multidrug efflux pump may also participate in eravacycline resistance and heteroresistance in *K*. *pneumoniae*.

## Materials and methods

### Bacterial strains and growth conditions

From January 2010 to December 2016, 393 nonduplicate *K*. *pneumoniae* isolates from various clinically sampled infections of individuals not previously exposed to tigecycline or eravacycline were collected from in-patients at Shenzhen Nanshan People’s Hospital at Shenzhen University School of Medicine in China. The strains were identified with a Phoenix 100 automated microbiology system (BD, Franklin Lakes, NJ, USA) and then, two subcultured generations were reidentified by matrix-assisted laser desorption ionization time-of-flight mass spectrometry (IVD MALDI Biotyper, Germany). All strains were cultured in Luria-Bertani medium at 37 °C. All procedures involving human participants were performed in accordance with the ethical standards of the Shenzhen University School of Medicine and with the 1964 Helsinki declaration and its later amendments. For this type of study, formal consent is not required.

### Antimicrobials

Two carbapenem drugs, namely imipenem (catalog no. MB1457) and meropenem (catalog no. MB1129), were purchased from Meilunbio (Dalian, China). The glycylcycline drug tigecycline (catalog no. E129449) was purchased from Aladdin (Shanghai, China). The novel fluorocycline drug eravacycline (catalog no. A13887-10) was purchased from AdooQ BioScience (Irvine, CA, USA).

### Antimicrobial susceptibility testing

The ESBL production of *K*. *pneumoniae* isolates was detected using the Phoenix 100 automated microbiology system. MICs for imipenem, meropenem, tigecycline, and eravacycline were determined by the agar dilution method, according to the Clinical and Laboratory Standards Institute (CLSI) guidelines (CLSI-M100-S26). The MIC breakpoints for carbapenem were defined according to CLSI-M100-S26. Isolates observed to have elevated MICs for carbapenem were confirmed by a manual ETEST® (bioMérieux) to have a reduced susceptibility to either imipenem or/and meropenem^29^.

### PAP analysis

PAPs were obtained as described previously^30^. One-hundred-microliter aliquots of a starting cell suspension (corresponding to a 0.5 McFarland standard for *K*. *pneumoniae* cultures grown on blood agar plates for 24 h at 37 °C; 1.0–1.5 × 10^8^ cfu/ml) were spread onto Mueller–Hinton agar plates with or without eravacycline (1, 2, 4, 6, 8, or 16 mg/l). After incubating for 24 h at 37 °C, the number of colonies was counted. As CLSI *Enterobacteriaceae* MIC breakpoints for tigecycline and eravacycline have not yet been established, *K*. *pneumoniae* isolates with an MIC ≥4 mg/l for tigecycline or eravacycline were considered to be not susceptible based on the reference studies^17,31^. Eravacycline heteroresistance was defined as the presence of eravacycline-susceptible isolates with an eravacycline MIC of <2 mg/l, in which detectable subpopulations grew in the presence of ≥4 mg/l eravacycline, with a detection limit of 20 cfu/ml. Each analysis was performed three times.

### Efflux inhibition

Efflux pump activity in tigecycline- and/or eravacycline-resistant *K*. *pneumoniae* isolates was detected using the efflux pump inhibitor (EPI) Phe-Arg-β-naphthylamide (PAβN, Sigma). Tigecycline and eravacycline MICs were determined by the agar dilution method in the presence and absence of PAβN (50 mg/l). Significant inhibition of efflux pumps was confirmed based on the MIC reduction to a quarter (or more) of the baseline values in the presence of EPI^8^.

### qRT-PCR

Transcript expression levels of the efflux pump genes *acrA*, *acrB*, *tolC*, *oqxA*, *oqxB*, *acrE*, *acrF*, *macA*, and *macB* and their transcriptional regulator genes *acrR*, *marA*, *soxS*, *rarA*, *robA*, and *ramA* were determined by qRT-PCR, with the primers listed in Table S4. Total bacterial RNA was extracted with an RNeasy mini kit (QIAGEN GmbH, Hilden, Germany), and cDNA was synthesized with a PrimeScript RT reagent kit (TAKARA BIO INC, Shiga, Japan). Finally, qRT-PCR was performed using a SYBR Premix Ex Taq II kit (TAKARA BIO INC, Shiga, Japan) in a Mastercycler ep realplex system (Eppendorf, Hamburg, Germany), with an initial incubation at 95 °C for 2 min, followed by 40 cycles of 15 s at 95 °C and 60 s at 60 °C. Each sample was run in triplicate. The expression of target genes was normalized relative to the 16S rRNA housekeeping gene *rrsE*. Threshold cycle (Ct) numbers were confirmed by the qRT-PCR system software, and data were analyzed in accordance with the 2^−ΔΔCt^ method. The expression levels of the target genes were compared with those of *K*. *pneumoniae* ATCC 13883 (tigecycline susceptible^9^ and eravacycline susceptible [according to our sensitivity test] strain, expression = 1).

### Detection of mutations in *acrR*, *ramR*, *rpsJ*, *oqxAB*, and *macAB*

The *acrR*, *ramR*, and *rpsJ* codons of the S10 ribosomal protein-encoding genes, as well as those of the efflux pump-encoding genes *oqxAB* and *macAB* were amplified by PCR (primers listed in Table S4) and sequenced. Mutations in *acrR*, *ramR*, and *rpsJ* in *K*. *pneumoniae* isolates were identified by comparison with a reference sequence, namely, the genome of *K*. *pneumoniae* subsp. *pneumoniae* MGH 78578 (GenBank accession number CP000647)^9^. Mutations in *oqxAB* and *macAB* were identified by comparison with reference sequences from the NCBI database (for detailed sequences see Tables S2 and S3 footnotes).

### Overexpression of OqxAB and MacAB in eravacycline-sensitive isolates of *K*. *pneumoniae*

Complete *oqxAB* gene was amplified by PCR from the eravacycline-heteroresistant clinical isolate EKP82-2, and the *macAB* gene was amplified from eravacycline-heteroresistant clinical isolate EKP129-1. The PCR fragments were purified and digested with endonucleases *Nhe*I and *Bgl*II and then were inserted into the plasmid pZP1137 for gene overexpression. Correct cloning was verified by PCR and sequencing. Verified plasmid constructs were introduced into three eravacycline-sensitive *K*. *pneumoniae* clinical isolates: CRKP9, EKP130, and EKP209. All strains, plasmids, and primers used for overexpression are listed in Tables S5 and S6. The overexpression of OqxAB and MacAB was induced with 0.2% arabinose (Ara). All assays were performed at least in triplicate.

### Statistical analysis

Continuous data are reported as the means ± standard deviations (SDs) and were analyzed with Student’s *t* tests, one-way factorial analyses of variance (ANOVA), or nonparametric Mann–Whitney *U* tests. Categorical data are reported as numbers (with percentages) and were compared with Chi-square or Fisher’s exact tests. *P* values <0.05 were regarded as significant. All data were analyzed in SPSS (version 17.0, Chicago, IL, USA).

## Electronic supplementary material

Figure S1

Figure S2

Figure S3

Figure S4

Supplementary Tables and Figures
