# Supplementary figures and images for: Overexpression of OqxAB and MacAB efflux pumps contributes to eravacycline resistance and heteroresistance in clinical isolates of Klebsiella pneumoniae
Source: Emerg Microbes Infect. 2018 Aug 1;7:139. doi: 10.1038/s41426-018-0141-y (PMC6070572; doi:10.1038/s41426-018-0141-y)

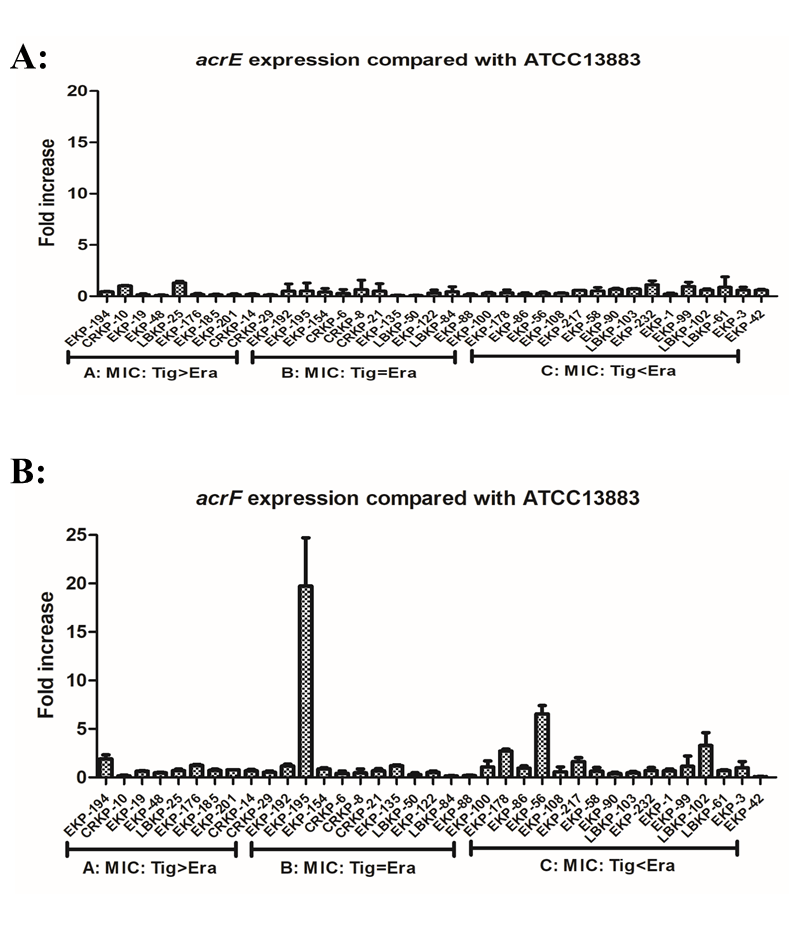

Supplement: Supplementary file 1 — Figure S1 [file 41426_2018_141_MOESM1_ESM.tif]

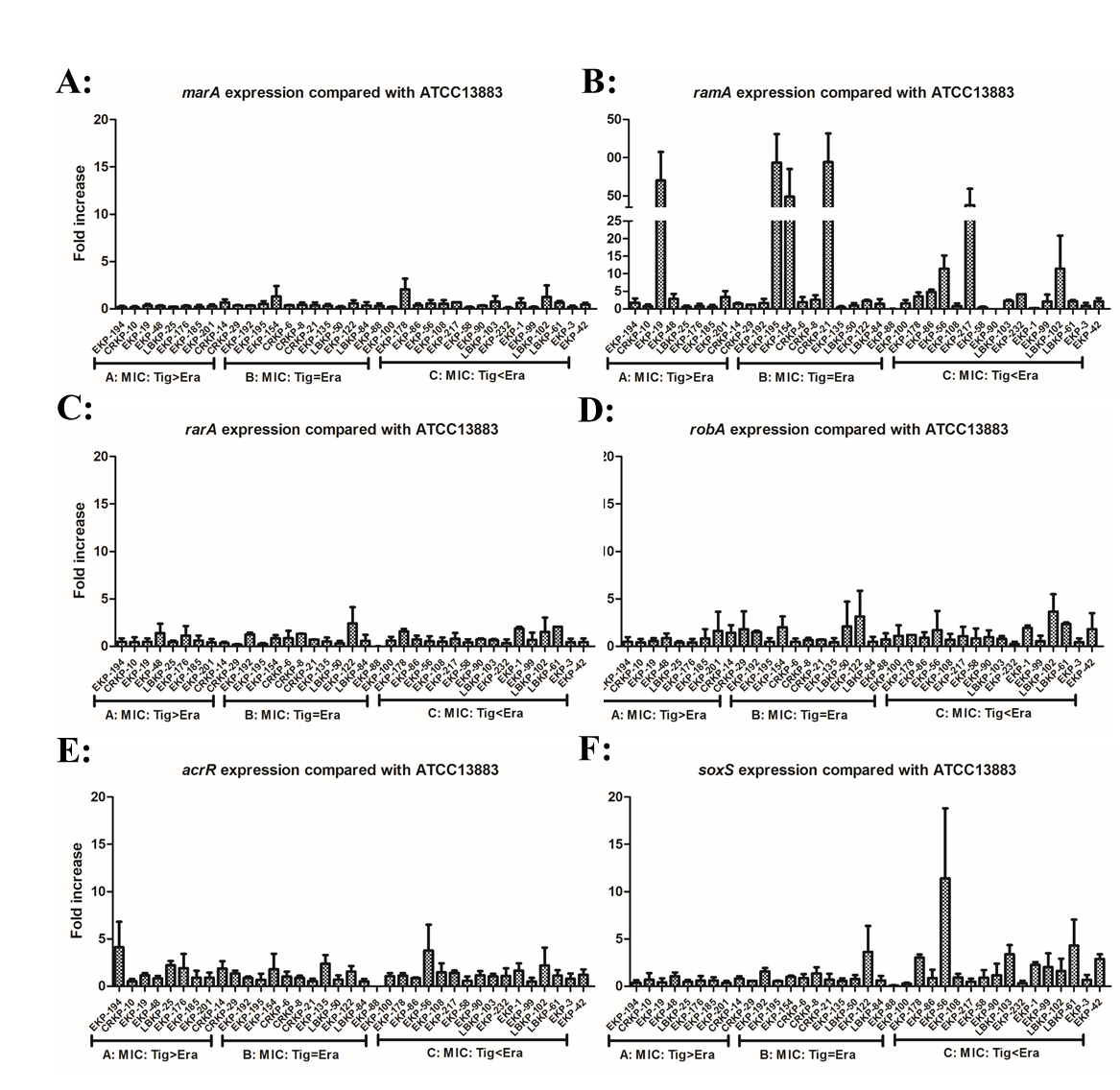

Supplement: Supplementary file 2 — Figure S2 [file 41426_2018_141_MOESM2_ESM.tif]

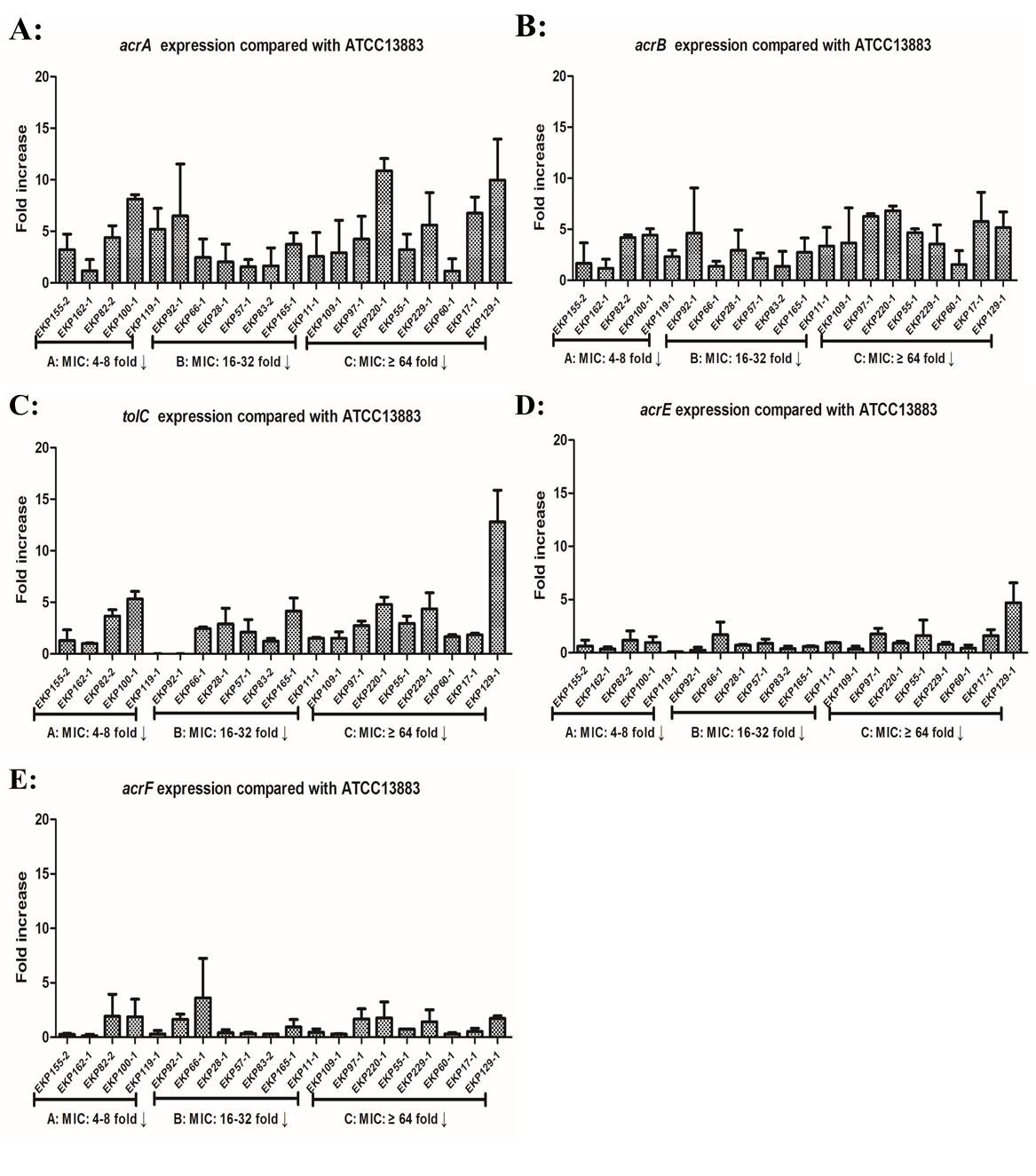

Supplement: Supplementary file 3 — Figure S3 [file 41426_2018_141_MOESM3_ESM.tif]

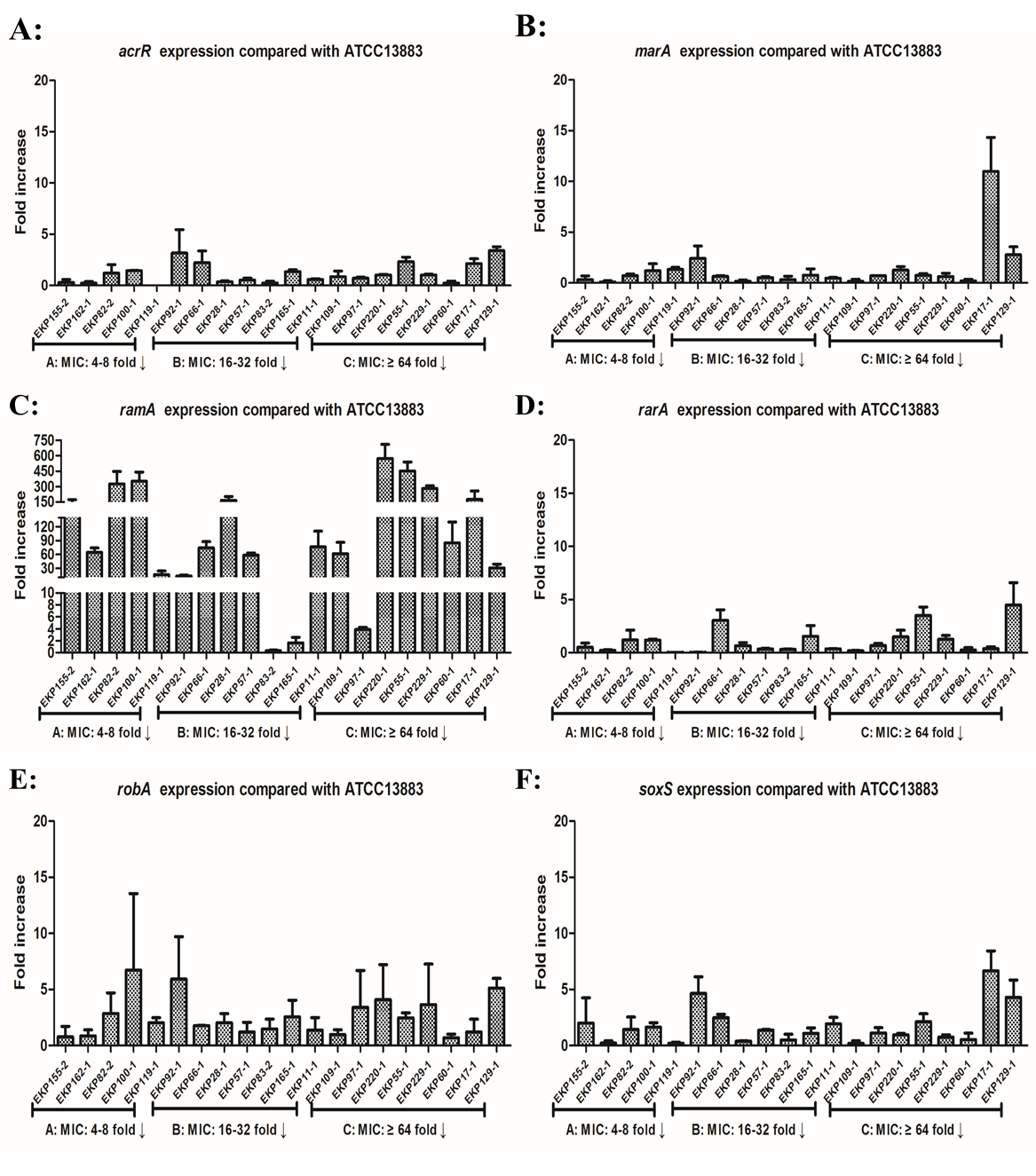

Supplement: Supplementary file 4 — Figure S4 [file 41426_2018_141_MOESM4_ESM.tif]
