# Supplementary material for: Overexpression of OqxAB and MacAB efflux pumps contributes to eravacycline resistance and heteroresistance in clinical isolates of Klebsiella pneumoniae
Source: Emerg Microbes Infect. 2018 Aug 1;7:139. doi: 10.1038/s41426-018-0141-y (PMC6070572; doi:10.1038/s41426-018-0141-y)
Supplement: Supplementary file 5 — Supplementary Tables and Figures [file 41426_2018_141_MOESM5_ESM.docx]

**Supplementary tables and figures:**

**Table S1.** Ribosomal protein gene mutations in 47 tigecycline- or eravacycline-sensitive clinical isolates of *K. pneumoniae*.

| **Isolates** | **MIC (mg/L)** | | **Local repressor gene mutation(s)** | | |
| --- | --- | --- | --- | --- | --- |
|  | **Tigecycline** | **Eravacycline** | ***acrR*** | ***rpsJ*** | ***ramR*** |
| 160730008 | 0.250 | 0.250 | - | - | - |
| 160901023 | 0.250 | 0.250 | - | - | D77H |
| 161028059 | 0.250 | 0.250 | - | - | - |
| CRKP-27 | 0.500 | 0.250 | P181L | - | - |
| CRKP-30 | 0.500 | 0.250 | - | - | - |
| CRKP-32 | 0.500 | 0.250 | - | - | - |
| CRKP-34 | 0.500 | 0.250 | - | - | - |
| CRKP-40 | 0.250 | 0.125 | - | - | L58Q |
| CRKP-49 | 0.500 | 0.250 | - | - | - |
| CRKP-54 | 0.500 | 0.250 | - | - | - |
| CRKP-56 | 0.500 | 0.250 | - | - | - |
| CRKP-59 | 0.500 | 0.250 | - | - | - |
| CRKP-62 | 0.500 | 0.250 | - | - | - |
| CRKP-63 | 0.500 | 0.250 | - | - | - |
| CRKP-9 | 0.500 | 0.250 | - | - | - |
| EKP-113 | 0.250 | 0.250 | - | - | - |
| EKP-117 | 0.250 | 0.250 | - | - | - |
| EKP-120 | 0.500 | 0.250 | - | - | - |
| EKP-130 | 0.500 | 0.250 | - | - | - |
| EKP-132 | 0.500 | 0.250 | - | - | - |
| EKP-138 | 0.250 | 0.250 | - | - | - |
| EKP-150 | 0.250 | 0.250 | L197P | - | - |
| EKP-169 | 0.250 | 0.250 | - | - | - |
| EKP-170 | 0.500 | 0.250 | - | - | - |
| EKP-171 | 0.500 | 0.250 | - | - | - |
| EKP-172 | 0.500 | 0.250 | - | - | - |
| EKP-173 | 0.500 | 0.250 | - | - | - |
| EKP-180 | 0.500 | 0.250 | - | - | - |
| EKP-200 | 0.500 | 0.250 | - | - | Y147N |
| EKP-209 | 0.125 | 0.250 | - | - | - |
| EKP-218 | 0.125 | 0.250 | - | - | - |
| EKP-227 | 0.125 | 0.250 | - | - | - |
| EKP-27 | 0.500 | 0.250 | - | - | - |
| EKP-34 | 0.125 | 0.250 | - | - | - |
| EKP-37 | 0.125 | 0.250 | - | - | - |
| EKP-40 | 0.125 | 0.250 | T144I | - | - |
| EKP-7 | 0.125 | 0.250 | - | - | - |
| EKP-87 | 0.500 | 0.250 | - | - | A19V |
| LBKP-14 | 0.250 | 0.250 | - | - | - |
| LBKP-29 | 0.500 | 0.250 | - | - | - |
| LBKP-35 | 0.500 | 0.250 | - | - | - |
| LBKP-4 | 0.500 | 0.250 | - | - | - |
| LBKP-47 | 0.500 | 0.250 | - | - | - |
| LBKP-71 | 0.250 | 0.250 | - | - | - |
| LBKP-74 | 0.250 | 0.250 | - | - | - |
| LBKP-76 | 0.500 | 0.250 | - | - | - |
| LBKP-93 | 0.250 | 0.250 | - | - | - |

**Table S2.** Mutations of *oqxAB* in these clinical isolates of *K. pneumoniae* with the overexpression of OqxAB efflux pumps (overexpressed the *oqxA* or *oqxB* >3-fold greater than ATCC 13883 reference strain).

| **Isolates** | **MIC (mg/L)** | | **Overexpression (-fold)^a^** | | **Mutations (amino acids)^b^** | |
| --- | --- | --- | --- | --- | --- | --- |
|  | **Era** | **Era+PAβN** | ***oqxA*** | ***oqxB*** | ***oqxA*** | ***oqxB*** |
| **Resistance** |  |  |  |  | **-** | **-** |
| EKP-195 | 16 | 1 | 2.84±0.41 | 4.71±1.44 | **-** | **-** |
| EKP-154 | 8 | 1 | 210.93±2.59 | 186.55±37.07 | **-** | **-** |
| EKP-135 | 4 | 0.5 | 137.54±108.87 | 52.34±12.62 | **-** | **-** |
| EKP-122 | 2 | 1 | 8.88±7.11 | 2.49±1.22 | **-** | **-** |
| LBKP-84 | 2 | 0.5 | 8.46±3.08 | 3.73±1.05 | **-** | **-** |
| EKP-178 | 16 | 1 | 35.64±3.61 | 61.60±12.05 | **-** | **-** |
| EKP-86 | 8 | 1 | 309.02±139.87 | 188.15±74.07 | **-** | **-** |
| EKP-108 | 8 | 1 | 223.57±49.67 | 192.07±21.43 | **-** | **-** |
| EKP-217 | 8 | 1 | 96.53±47.67 | 114.89±9.21 | **-** | **-** |
| EKP-1 | 4 | 1 | 145.01±57.21 | 106.31±5.83 | **-** | **-** |
| LBKP-102 | 4 | 1 | 290.61±94.05 | 145.34±15.78 | **-** | **-** |
| LBKP-61 | 4 | 1 | 168.84±96.24 | 150.49±14.73 | **-** | **-** |
| **Heteroresistance** |  |  |  |  | **-** | **-** |
| EKP82-2 | 16 | 2 | 17.89±1.42 | 8.42±1.64 | **-** | H244L, E248K |
| EKP100-1 | 16 | 2 | 15.18±4.17 | 4.13±1.18 | **-** | - |
| EKP119-1 | 8 | 1 | 16.51±3.75 | 2.12±0.68 | D242N | - |
| EKP92-1 | 16 | 1 | 7.81±5.07 | 3.47±2.02 | - | - |
| EKP28-1 | 8 | 0.5 | 5.89±1.98 | 5.28±2.87 | - | - |
| EKP57-1 | 8 | 0.5 | 121.16±62.59 | 296.34±114.65 | - | - |
| EKP83-2 | 16 | 0.5 | 10.46±3.78 | 29.28±7.78 | - | - |
| EKP165-1 | 16 | 0.5 | 10.36±2.09 | 4.98±0.34 | - | - |
| EKP97-1 | 16 | 0.25 | 6.43±4.22 | 15.14±1.78 | - | - |
| EKP220-1 | 16 | 0.25 | 36.74±6.53 | 14.13±0.62 | - | - |
| EKP229-1 | 8 | 0.125 | 12.55±3.33 | 5.96±3.29 | - | - |
| EKP17-1 | 16 | 0.125 | 14.70±2.31 | 12.22±4.81 | - | - |
| EKP129-1 | 16 | 0.125 | 17.54±2.86 | 26.85±2.79 | - | - |

Era: Eravacycline; PAβN, Phe-Arg-b-naphthylamide (50mg/L); **^a^**: overexpressed fold greater than tigecycline-susceptible K. pneumoniae ATCC 13883 reference strain, mean±SD;

**^b^**: **OqxA reference sequences (NCBI numbers):** WP_071570989.1, WP_002914189.1, WP_080816181.1, [WP_012540822.1](https://www.ncbi.nlm.nih.gov/protein/WP_012540822.1/), WP_004174785.1, WP_087528385.1, WP_050885831.1, WP_064171164.1, WP_023342074.1, WP_046042626.1, WP_040170192.1, WP_094986461.1, WP_060415812.1, WP_047693838.1, WP_040088492.1, WP_102016067.1, WP_073901188.1, WP_087742862.1, WP_048290002.1, WP_023284143.1, WP_082226006.1, WP_080923024.1, WP_064178858.1, WP_048253754.1, WP_040150752.1.

**OqxB reference sequences (NCBI numbers):** WP_012540821.1, WP_076027157.1, WP_095123809.1, WP_064323814.1, WP_032420468.1, WP_061153613.1, WP_040241524.1, PXI72095.1, WP_087791584.1, WP_085858220.1, WP_048976156.1, WP_040250583.1, WP_064173758.1, WP_046042624.1, WP_023286794.1, WP_104190268.1, WP_064156110.1, WP_087850748.1, WP_080897721.1, WP_065809338.1, WP_040172527.1, WP_104457225.1, WP_040189683.1, WP_038432953.1, WP_109233571.1, WP_082226007.1, WP_064839466.1, WP_032444709.1, WP_094315951.1, WP_048299610.1, WP_032415399.1.

**Table S3.** Mutations of *macAB* in these clinical isolates of *K. pneumoniae* with the overexpression of MacAB efflux pumps (overexpressed the *macA* or *macB* >3-fold greater than ATCC 13883 reference strain).

| **Isolates** | **MIC (mg/L)** | | **Overexpression (-fold)^a^** | | **Mutations (amino acids)^b^** | |
| --- | --- | --- | --- | --- | --- | --- |
|  | **Era** | **Era+PAβN** | ***macA*** | ***macB*** | ***macA*** | ***macB*** |
| **Resistance** |  |  |  |  |  |  |
| CRKP-8 | 8 | 0.5 | 1.52±0.21 | 4.78±2.66 | - | - |
| CRKP-21 | 8 | 0.5 | 3.61±2.42 | 0.98±0.35 | - | - |
| **Heteroresistance** |  |  |  |  |  |  |
| EKP82-2 | 16 | 2 | 13.78±9.01 | 3.73±1.24 | - | I216F |
| EKP100-1 | 16 | 2 | 19.22±22.69 | 11.65±0.31 | - | - |
| EKP92-1 | 16 | 1 | 0.005±0.0.003 | 4.07±0.35 | - | - |
| EKP66-1 | 16 | 1 | 12.07±7.18 | 3.36±0.09 | - | - |
| EKP28-1 | 8 | 0.5 | 4.76±2.05 | 3.95±2.01 | - | - |
| EKP165-1 | 16 | 0.5 | 10.20±8.04 | 7.10±2.37 | - | - |
| EKP220-1 | 16 | 0.25 | 18.85±15.86 | 5.09±3.55 | - | - |
| EKP55-1 | 16 | 0.25 | 13.36±7.74 | 2.27±0.73 | - | - |
| EKP229-1 | 8 | 0.125 | 28.35±20.46 | 13.68±0.70 | - | - |
| EKP129-1 | 16 | 0.125 | 21.85±8.51 | 5.74±1.56 | - | - |

Era: Eravacycline; PAβN, Phe-Arg-b-naphthylamide (50 mg/L); **^a^**: overexpressed fold greater than tigecycline-susceptible *K. pneumoniae* ATCC 13883 reference strain, mean±SD;

**^b^: MacA reference sequences (NCBI numbers)**: NC_016845.1, WP_071556640.1, WP_065874941.1, WP_039108682.1, WP_087637889.1,WP_080893439.1,WP_040169856.1,WP_101516549.1,WP_102099642.1,WP_101862887.1,WP_064146978.1,WP_040088746.1,WP_110097744.1,WP_109223397.1,WP_102787758.1,WP_071080485.1,WP_040241688.1,PXJ55937.1,WP_046622746.1,PXH72743.1,WP_064189330.1,WP_065812160.1,WP_109910267.1,WP_047666229.1,WP_064180367.1,WP_070181425.1;

**MacB reference sequences (NCBI numbers)**: NC_009648.1, NC_011283.1, WP_060876116.1, WP_048293411.1, WP_064180366.1, WP_046882271.1, WP_065898358.1, WP_080837446.1, PXI71081.1, WP_109226283.1, WP_087872490.1, WP_064150402.1, WP_109266629.1, WP_103517476.1, WP_101973190.1, WP_080841188.1, WP_058330789.1, WP_104468262.1, WP_065928333.1, WP_064161764.1, WP_012068502.1, WP_049117877.1, WP_004209695.1

**Table S4.** Primers used for qRT-PCR and PCR amplification.

| **Target** | **Primer** | **Sequence (5'-3')** | **Purpose** | **Source or Ref.** |
| --- | --- | --- | --- | --- |
| ***rrsE*** | rrsE-F | CTACAATGGCATATACAA | qRT-PCR | This study |
|  | rrsE -R | TTCTGATCTACGATTACT |  |  |
| ***acrA*** | acrA-F | GGCAAACATGGATCAACTG | qRT-PCR | This study |
|  | acrA-R | GGCGGTATCGTAGTCTTG |  |  |
| ***acrB*** | acrB-F | GGAAGATACACCGCAGTT | qRT-PCR | This study |
|  | acrB-R | TGTTAATGTCGCTGATGGA |  |  |
| ***tolC*** | tolC-F | CTACGCTGTATAACGCTAA | qRT-PCR | This study |
|  | tolC-R | CTAACGCCGACTTAATGT |  |  |
| ***oqxA*** | oqxA-F | CGCAGCTTAACCTCGACTTCA | qRT-PCR | 19 |
|  | oqxA-R | ACACCGTCTTCTGCGAGACC |  |  |
| ***oqxB*** | oqxB-F | ATCAGGCGCAGGTTCAGGT | qRT-PCR | 19 |
|  | oqxB-R | CGCCAGCTCATCCTTCACTT |  |  |
| ***acrE*** | acrE-F | CCCATTTGACGGTGAAAC | qRT-PCR | This study |
|  | acrE-R | ACTGCCTGATCGTATTCC |  |  |
| ***acrF*** | acrF-F | GAGCGACATTAACAACAT | qRT-PCR | This study |
|  | acrF-R | TATATCCAGCGTGAAGTG |  |  |
| ***macA*** | macA-F | TATGAAGGTAAACTGAAAGACA | qRT-PCR | This study |
|  | macA-R | GAAGCGGGCATAATAGAA |  |  |
| ***macB*** | macB-F | TGAAGGTTATGACAGTGA | qRT-PCR | This study |
|  | macB-R | TCCAGGTGAATACATCTT |  |  |
| ***acrR*** | acrR-F | TCCACTCTCAGTTATCAG | qRT-PCR | This study |
|  | acrR-R | GACGAACTCACACTTATG |  |  |
| ***marA*** | marA-F | ATGATGTCCAGACGTAATAATGA | qRT-PCR | This study |
|  | marA-R | GGCGATTCCAGGTTATCC |  |  |
| ***ramA*** | ramA-F | GCTGCGTATTGATGATAT | qRT-PCR | This study |
|  | ramA-R | TCTCCCTTGTACTGTAAA |  |  |
| ***rarA*** | rarA-F | GACCATCCTGTTTGTTGAC | qRT-PCR | This study |
|  | rarA-R | GTGCCGTCTTCAATATGC |  |  |
| ***robA*** | robA-F | TATTCTATACCACCGCGCTGAC | qRT-PCR | 19 |
|  | robA-R | GTGCCGTAGACGGTCAGGAT |  |  |
| ***soxS*** | soxS-F | CTTAACATTGATATAGTCGCCAGA | qRT-PCR | This study |
|  | soxS-R | CATCACGGTACGGAACATC |  |  |
| ***acrR*** | acrR-F | ACGTAACCTCTGTAAAGTCAT | Mutation  detection | 9 |
|  | acrR-R | TTAAGCTGACAAGCTCTCCG |  |  |
| ***ramR*** | ramR-F | AGTGTTTCCGGCGTCATTAG | Mutation  detection | 9 |
|  | ramR-R | AGTCGTCAAGACGATTTTCAATTTT |  |  |
| ***rpsJ*** | rpsJ-F | AGTAACGCGGTTTGCTTC | Mutation  detection | 9 |
|  | rpsJ-R | ACAGCCGGTTCGATATGA |  |  |
| ***oqxA*** | MoqxA-F | GCGATGATCGACACAAATGG | Mutation  detection | This study |
|  | MoqxA-R | ACGTCCGGATATTCGCTCAC |  |  |
| ***oqxB*** | MoqxB-F | CTCAACTGATCCCTAATCAG | Mutation  detection | This study |
|  | MoqxB-R | GATGAAGCAGAGCAGGCTTC |  |  |
| ***macA*** | MmacA-F | ATGGGTGTCCTGTTGCCAAC | Mutation  detection | This study |
|  | MmacA-R | CTGCGACGTATATCTCGCAG |  |  |
| ***macB*** | MmacB-F | ATGAGGGAGATGAAGTGATCG | Mutation  detection | This study |
|  | MmacB-R | GCTTCATTGTGTACATCCTGC |  |  |

RT-PCR, quantitative reverse transcription polymerase chain reaction

**Table S5.** Strains and plasmids used for the overexpression of *K. pneumoniae* OqxAB and MacAB.

| **Strain or plasmid** | **Antibiotic resistance^a^** | **Description** | **Source** |
| --- | --- | --- | --- |
| ***K. pneumoniae* strain** |  |  |  |
| CRKP9 | - | clinical isolate, tigecycline and eravacycline sensitive | This study |
| EKP130 | - | clinical isolate, tigecycline and eravacycline sensitive | This study |
| EKP209 | - | clinical isolate, tigecycline and eravacycline sensitive | This study |
| **Plasmids** |  |  |  |
| pZP1136 | Amp^100^ | Arabinose-inducible, myc tag, pBR322 ori, ampR | This study |
| pZP1137 | Amp^100^, Kan^50^ | pZP1136 containing a 1.2-kb kanamycin resistance fragment from pET28a | This study |
| pZP1137-*oqxAB* | Amp^100^, Kan^50^ | pZP1137 with insertion of the gene *oqxAB*, for OqxAB expression | This study |
| pZP1137-*macAB* | Amp^100^, Kan^50^ | pZP1137 with insertion of the gene *macAB*, for MacAB expression | This study |

**^a^**: Superscripts indicate the concentrations of antibiotics (in µg/ml) to which the plasmids are resistant; Kan, kanamycin; Amp, ampicillin.

**Table S6.** PCR primers used for the overexpression of *K. pneumoniae* OqxAB and MacAB.

| **Primers** | **Sequence (5' →3' )** | **Purpose** | **Annotation^a^** |
| --- | --- | --- | --- |
| Kan-F | CCCAAGCTTGAGTCCAACCCGGTAAGACAC | Amplification of kanamycin resistance fragment from pET28a | HindIII |
| Kan-R | AAAAGTACTAGAAAAACTCATCGAGCAT |  | ScaI |
| GoqxAB-F | CTAGCTAGCTACCGGAATAAAAATAATGAGCCTGCAAAAAACCTGG | Amplification of *oqxAB* gene | NheI |
| GoqxAB-R | GGAAGATCTCTAGGCGGGCAGATCCTCCTGGACCGGCTTC |  | BglII |
| JoqxAB-F | ACCTGATTGGCGATGCCGATC | Screening recombinant plasmid of pZP1137-*oqxAB* |  |
| JoqxAB-R | AACAGGAATCGAATGCAACCG |  |  |
| GmacAB-F | CTAGCTAGCAGCAGGAATCGGAATATGAAAGTGAAGGGAAAACG | Amplification of *macAB* gene | NheI |
| GmacAB-R | GGAAGATCTCTATTCTCGCGCCAGGGCATCCACCGGATCGA |  | BglII |
| JmacAB-F | ATGCTGGTGTCGGTGACCGAA | Screening recombinant plasmid of pZP1137-*macAB* |  |
| JmacAB-R | GAACACTGCCAGCGCATCAAC |  |  |

**^a^**Underlined sequences represent the restriction enzyme sites of primers;

**Supplementary Figure legends:**

**Figure S1.** Relative gene expression, expressed as fold change, of *acrEF* efflux pumps in 37 clinical isolates of *K. pneumoniae*. Expression levels were detected by qRT-PCR, with tigecycline-susceptible *K. pneumoniae* ATCC 13883 as the reference strain (expression = 1).

**Figure S2.** Relative gene expression, expressed as fold change, of *acrR*, *marA*,*ramA*, *rarA,robA* and *soxS* transcriptional regulators in 37 clinical isolates of *K. pneumoniae*. Expression levels were detected by qRT-PCR, with tigecycline-susceptible *K. pneumoniae* ATCC 13883 as the reference strain (expression = 1).

**Figure S3.** Relative gene expression, expressed as fold change, of *acrAB-tolC* and *acrEF* efflux pumps in 20 clinical isolates of eravacycline heteroresistant *K. pneumoniae*. Expression levels were detected by qRT-PCR, with tigecycline-susceptible *K. pneumoniae* ATCC 13883 as the reference strain (expression = 1). MIC: 4–8-fold↓, eravacycline MICs decreased 4–8-fold in the presence of PAβN compared to eravacycline alone.

**Figure S4.** Relative gene expression, expressed as fold change, of *acrR*, *marA*, *ramA*, *rarA, robA* and *soxS* transcriptional regulators in 20 clinical isolates of eravacycline heteroresistant *K. pneumoniae*. Expression levels were detected by qRT-PCR, with tigecycline-susceptible *K. pneumoniae* ATCC 13883 as the reference strain (expression = 1). MIC: 4–8-fold↓, eravacycline MICs decreased 4–8-fold in the presence of PAβN compared to eravacycline alone.
